# Challenges, Barriers, Strategies Implementing an Oral Hygiene Program in Assisted Living Facilities Post COVID-19

**DOI:** 10.1093/geroni/igaa057.3426

**Published:** 2020-12-16

**Authors:** Kim Attanasi, Victoria Raveis

**Affiliations:** New York University, Maplewood, New Jersey, United States

## Abstract

[Objective] Almost 8% of the U.S. population, 65 and older, reside in long term care facilities with limited delivery of essential dental care to prevent and manage oral health disease. By 2050, this population is expected to increase by 1.6 billion. Multiple bi-directional connections exist between oral disease and overall health. [Methods] Faculty from the Dental Hygiene Department, New York University College of Dentistry conducted an extensive outreach effort and randomly selected assisted living facilities. Facilities were offered the opportunity to receive at no-cost, a dental hygiene-led, educational, preventive oral health program delivered virtually to their residents as a community service. Incentives discussed. [Results] Twenty-one facilities were contacted, 17 (94.4%) had no oral healthcare program; one had an oral health component. In 13 (72%), the concierge functioned as gatekeeper, unwilling to transfer calls or deliver messages. In five (28%), calls were directed to the activity coordinator. Feasibility concerns and uncertainty about oral health service necessity and resident safety were voiced. Two facilities mentioned familiarity with dental hygiene professionals. Strategic changes in outreach resulted in successfully engaging with facility administrators. Strategies included identifying directors with familiarity or experience with dental hygiene profession, establishing a portfolio and utilizing technology that facilitate incorporating COVID-19 protocols. [Conclusions] Efforts to initiate a dental hygiene-led virtual oral health program encountered gatekeeper challenges. Although facility activity coordinators acknowledged benefits for their population, they were not final decision-makers. It was necessary to implement strategies that facilitated discussing the virtual oral hygiene program directly with the facility’s executive leadership.

